# Destructive Role of COVID-19 Fear on Nurses Performance: Mediating Role of Stress

**DOI:** 10.3390/nursrep11040087

**Published:** 2021-11-24

**Authors:** Zahid Yousaf, Abdelmohsen A. Nassani, Mohamed Haffar

**Affiliations:** 1 Higher Education Department, Government College of Management Sciences, Mansehra 21300, Pakistan; 2 Department of Management, College of Business Administration, King Saud University, P.O. Box 71115, Riyadh 11587, Saudi Arabia; Nassani@ksu.edu.sa; 3 Department of Management, Birmingham Business School, Birmingham B15 2TT, UK; m.haffar@bham.ac.uk

**Keywords:** COVID-19 fear, COVID-19 stress, health care performance, health care sector

## Abstract

Given its importance to psychological issues, the COVID-19 pandemic has created numerous challenges for all individuals, but healthcare professionals and particularly nursing staff are at front lines, and their performance is significantly affected. The current study relates COVID-19 fear with psychological strain, i.e., stress amongst the nursing staff. Moreover, the intervening role of COVID-19 stress between COVID-19 fear and the performance of the nursing staff has also been tested. An online survey was conducted to collect data from nurses. A total of 471 responses of nurses were received during the process of online data collection from 16 November 2020 to 30 April 2021. Results revealed the significant effect of COVID-19 fear on COVID-19 stress and the performance of nurses. Additionally, the results substantiate that COVID-19 stress mediates between COVID-19 fear and the health care performance of nurses. COVID-19 fear has become a psychological consequence that increases stress among nursing staffs. This study fills the research gap about the performance of the health care sectors, particularly with respect to COVID-19 fear and COVID-19 stress among nurses. Hence, COVID-19 fear plays a significant role in COVID-19 stress in terms of influencing the health care performance of nurses. Overall, the results give pragmatic insights for the consequences of the COVID-19 pandemic.

## 1. Introduction

The performance of health care organizations is largely based on the interdependent work of their staffs and, particularly, embedded in the work efficiency of the nursing staff [1]. Existing studies have highlighted various factors that affect the work efficiency of the nursing staff [2]. In Pakistan, like the other developing countries, nursing staffs are facing several problems, such as workplace violence [3], long working hours [4], inflexible work schedules [5], work-family conflict [6], low salaries [7], and heavy workloads [8]. All of these issues are negatively affecting the health care performance of nurses by causing poor health [9], depression, anxiety and stress [10].

Recently, the pandemic circumstances emerged as one of the influencing factors that created far-reaching effects on the psychological aspects of all individuals [11]. The emergence of COVID-19 has been identified as a key psychological factor behind many health-related issues around the world. The COVID-19 pandemic is faced by both the developed as well as developing countries [12]. The most important consequence of COVID-19 is fear that influences the individual patterns of thinking, motivations, aspirations and behavior. COVID-19 fear interrupts the work role of employees in almost all kinds of organization [13]. According to Notara et al., the nursing staff plays a dynamic role in the improvement of the healthcare sector [14]. It is self-evident that COVID-19 pandemic fear extensively distracts from the performance of the nursing staff [15], ultimately influencing healthcare performance. Overall, the performance of the health care sector decreases due to the fear of COVID-19. 

Although COVID-19 fear is considered as a major an influencing factor in terms of the health care performance of nursing staff [16], COVID-19 fear is critical but not only the sole factor for the destruction of nursing staff performance. Consequently, the intention of this study was to highlight this research gap and explore the intervening role of stress among nursing staff in this link. Researchers have explored various factors that play an intervening role in this relationship e.g., [16,17,18]. This study also adds to the existing literature through the indirect effect of stress. Therefore, in contrast to previous studies, we contend that the inclusion of the mediating role of stress provides new insight into this relationship. Existing studies have discussed stress as one of the influential factors that increase the task distraction of health care professionals. Recently, health care professionals e.g., nursing staffs, scored higher on measures of stress due to the COVID-19 pandemic. COVID-19 fear is the fundamental force for the creation of stress [18]. External pressures exercised by the COVID-19 pandemic become the cause of stress among nurses [19]. Past studies have discussed that COVID-19 fear is one of the main drivers increasing stress among nursing staffs [20], which in turn has a negative impact on the healthcare performance of nurses. The health care performance of the nursing staff becomes necessary for health care sectors to respond positively and satisfactorily in a pandemic situation [21]. Higher levels of stress among health care professionals prevents them from executing the requirements of their job-related activities efficiently and effectively, which playsa destructive role with regard to the performance of these professionals, particularly the performance of the nursing staff.

The current study was conducted to identify the connection between COVID-19 fear, stress, and the health care performance of nurses. This study analyzed the role of COVID-19 fear for the cause of stress among the nursing staff, which in turn reduces the health care performance of these professionals. This study would be helpful for the health care sector management to respond to the COVID-19 pandemic in order to enhance health care performance of their nursing staff. Firstly, the current study examines the destructive role of COVID-19 fear on the health care performance of nurses. Secondly, it observes the outcome of COVID-19 fear on stress. Finally, it also highlights the intervening effect of stress between COVID-19 fear and performance of nurse’s connection. 

## 2. Materials and Methods

### 2.1. COVID-19 Fear and Health Care Performance of Nurses

At present, the consequences of the COVID-19 pandemic are, and the psychological effects of COVID-19 have also received great attention from academics and researchers [22]. The role of the health care sector is immense in this pandemic all over the world; therefore, there is dire need to explore the association between COVID-19 fear and performance of nursing staffs. Existing studies have revealed that the COVID-19 pandemic had a negative effect on the psychological well-being of health care professionals worldwide [23]. Psychological health issues are the leading obstacle to health care performance. Psychological issues can affect health care professionals’ motivation, concentration, and social interactions—crucial factors for health care professionals’ to perform successfully [24]. The COVID-19 pandemic, due to radical change it has caused to lifestyle, has badly affected the psychological health of the people. Quarantines and the freezing of social activities have created many stressors like fear and anxiety in the population. A recent study also shows that COVID-19 results in more pressures on health care sectors [25]. These pressures, such as overwork and staff shortages, along with COVID-19 fear, create challenges which limit the attention required to be paid to everyday tasks [26]. Less attention on work activities restricts health care professionals, such as nursing staffs, to perform their duties in the workplace, which ultimately reduces the performance of the health care sector.

**Hypothesis** **1** **(H1).** *COVID-19 fear is negatively related with performance of nurses.*

### 2.2. COVID-19 Fear and Stress

The COVID-19 pandemic is causing higher work burdens on nursing staffs that create psychological strain. This type of strain as a result of COVID-19 fear becomes a source of stress among nurses [27]. Stress due to the rescheduling of duties has been increased among nurses [28]. The fear of getting infected has made it compulsory to ensure social distancing, which has increased distance from friends and families, thus causing depression and stress [29]. Extra workload due to the COVID-19 pandemic is being faced by the health care workers, therefore adding to their stress. Extra burden and fatigue have created stress among nursing staffs [30]. Hence, it is proposed that:

**Hypothesis** **2** **(H2).** *COVID-19 fear and stress are positively related.*

### 2.3. Mediating Role of Stress between COVID-19 Fear and Health Care Performance

It is self-evident that the consequences of the COVID-19 pandemic have shown that fear and stress have significant effectson the health and performance of individuals. Most of the studies e.g., [16,31] have found a significant correlation between COVID-19 fear and workplace performance. COVID-19 fear is an important factor for reducing the performance, but not a sole factor for declining individual performance. COVID-19 fear is not only directly related to individual performance but also hasan indirect effect through intervening variables. Therefore, in the current study we proposed that stress is a potential intervening variable between COVID-19 fear and health care performance of nursing staffs. Stress is defined and used by researchers as a psychological strain due to the occurrence of some unusual event or occurrence [29]. Due to stress, health care professionals have less concentration and motivation for the performance of tasks in the workplace [32]. On the other hand, COVID-19 fears among nursing staffs causes stress [27], which ultimately reduces the performance of the nursing staff.

In the current study we developed arguments that stress among nursing staff due to COVID-19 fear playa mediating role for COVID-19 and the performance of nurses. However, the mediating role of stress in the relationship between COVID-19 fear and health care performance of the nursing staff is still insufficiently explored. Overall, researchers have great attention towards the psychological consequences of the COVID-19 pandemic among the nursing staff. However, the association between COVID-19 fear and performance among nursing staff in Pakistan remain unexplored. On the other hand, the role of stress as a mediator is still ignored. In the current study, we try to fill this gap, and used data that was collected from nurses in order to explore the connection between COVID-19 fear and heath care performance of nurses with the intervening role of stress in Pakistan.

**Hypothesis** **3** **(H3).** *Stress is negatively associated with performance of nurses.*

**Hypothesis** **4** **(H4).** *Stress mediate between COVID-19 fear and performance link.*

### 2.4. Study Design, Setting and Participants

A cross-sectional approach was implemented as a study plan. In the current study, self-administrated questionnaire techniques were used in data-collection and for testing the study hypotheses to reach empirical findings. Before distribution of questionnaires to the respondents, the experts from industry and academia checked the language and the questions of the survey. Validation of study constructs was obtained from the experts and academia. Furthermore, for the completion of current research, we followed the national as well as international standards of conducting research. 

The target population for the current study consists of hospitals. In January 2021, 53 hospitals were identified. Prior permission has been taken in the form of written approvals from the directors of the health care departments’ of hospitals. Selected hospitals were asked to complete informed consent forms. On-line surveys were conducted to receive responses from the health care professionals between 16 November, 2020 and 30 April 2021.

The target population for the current study consists of health care staff of various hospitals. For the current study, registered health care staff of 53 hospitals was chosen as the research population. The sample size was 1070 with a confidence level of 95% and margin of error of 5%. For the purpose of sampling we obtained the staff lists from 53 hospitals along with their email addresses. Specifically, Excel software was utilized in order to specify respondents from care staff. The staff was then ordered on the basis of random numbers. Four research associates were hired to approach and brief the sampled staff. A short training was provided to the research associates regarding the online data collection process and purpose of the research. Only the nursing staff members with permanent and full-time jobs were included. The detail of Supplementary Material is also available online.

### 2.5. Study Measures

#### 2.5.1. Dependent Variable

The health care performance of nurses is used as a dependent variable. The emergence of the COVID-19 pandemic dramatically changes the working environment of all kinds of organizations. Therefore, the performance of professionals becomes of greater attention to researchers and academics as a result of the COVID-19 pandemic. The current study used the performance of nursing staff as a dependent variable. The health care performance of nurses was measured with 13 items formulated and validated by Oswald [33]. Appendix A shows details of questions.

#### 2.5.2. Independent Variable

The emergence of the COVID-19 pandemic influences all individuals worldwide in respect to their physical, mental and psychological health. The current study used COVID-19 fear as an independent variable. The COVID-19 fear measure with 07 items was formulated and validated by [11].

#### 2.5.3. Mediating Variable

COVID-19 stress is used as a mediating variable. The seven items measured were used for the measurement of COVID-19 stress, as formulated and develop by Zurlo et al. [34], based on transactional models of stress proposed by Lazarus and Folkman [35].

### 2.6. Methods

In the current study data analysis was performed with the help of various statistical techniques such as such as descriptive statistics, correlation, regression and structural equation modeling (SEM).

## 3. Results

### 3.1. Descriptive Statistics

The findings of descriptive and correlation analysis wareshown in Table 1. The results of descriptive statistics stated that health care performance gains higher value reported by average respondent i.e., 3.9. Furthermore, COVID-19 fear also indicated a higher value of respondent sample means i.e., 3.8. The respondent sample means for COVID-19 stress was 3.5. 

The analysis of correlations has shown significant positive association among study constructs. Table 1 also contains the statistics of correlation among COVID-19 fear, COVID-19 stress and the health care performance of nurses. Results revealed negative connection between COVID-19 fear and the health care performance of nurses (*r* = −0.21), the positive association between COVID-19 fear and COVID-19 stress (*r* = 0.32), and a negative relationship between COVID-19 stress and the health care performance of nurses (*r* = −0.30).

### 3.2. Confirmatory Factor Analysis

CFA, one of the mechanisms used in SEM, was applied to the confirmation of constructs discriminant validity. Three separate models were run and verified with diverse configurations. The first model was based on a single-factor in which we combined COVID-19 fear, COVID-19 stress and the health care performance of nurses. The second model’s formation contains two-factors; COVID-19 fear and COVID-19 stress merge as a one factor, with thehealth care performance of nurses as a second one. Lastly, the third model involved four separate factors, i.e., COVID-19 fear, COVID-19 stress and health care performance of nurses. According to the finding generated with the help of CFA analysis, the good fit of the model was confirmed with three separate factors i.e., (χ^2^ = 336.19; *df* = 744 *p* < 0.001; CFI = 0.92; GFI = 0.94 and SRMR = 0.058). These findings met the criteria suggested by Kyriazos [36]. Table 2 presents the outcomes of reliability statistics for the study constructs.

### 3.3. Testing Direct Effects

With the help of regression analysis we tested the direct relationships of COVID-19 fear and the health care performance of nurses. Furthermore, the direct effects of COVID-19 fear on COVID-19 stress. Finally, the direct association between COVID-19 stress and health care performance of nurses was also tested with the help of regression coefficients. The results presented in Table 3 present direct effects. Model 1 and 2 of Table 3 contain the coefficients of direct effect of COVID-19 fear, COVID-19 stress and the health care performance of nurses. Moreover, Models 3 and 4 contain the results of the direct effect of COVID-19 fear on COVID-19 stress. 

Model 1 confirmed the study Hypothesis 1 on the basis of the results i.e., negative direct association between COVID-19 fear and the health care performance of nurses. The findings shown in Model 1 revealed that COVID-19 fear negatively predicted the health care performance of nurses (*β* = −0.23**). On the basis of these findings study Hypothesis 1 is confirmed. 

Model 4 contains the coefficient of direct effect of COVID-19 fear on COVID-19 stress. COVID-19 fear positively predicted COVID-19 stress (*β* = 0.34**). On the basis of these findings, study Hypothesis 2 is confirmed. Model 2 contains the coefficient for the direct effect of COVID-19 fear on the health care performance of nurses. The results also confirmed that COVID-19 fear significantly and negatively predicted the health care performance of nurses (*β* = −0.37**). Hence, study Hypothesis 3 is confirmed. Results presented in Model 2 also explain the intervening role of COVID-19 stress between COVID-19 fear and the health care performance of nurses. After COVID-19 stress is added, the coefficient of COVID-19 fear becomes insignificant (*β* = −0.37**) and (*β* = −0.13). The results of Model 2 confirmed Hypothesis 4 and show full mediation of COVID-19 stress between COVID-19 fear and the link to the health care performance of nurses.

## 4. Discussion

The primary objective of this research was to understand how COVID-19 fear reduces the performance of nurses via COVID-19 stress. For this purpose, the current study interlinked the COVID-19 fear with COVID-19 stress and the health care performance of nurses. Moreover, the intervening role of COVID-19 stress on the connection between COVID-19 fear and health care performance of nurses has also been tested. 

For the purpose of achieving the study objectives, we have formulated four hypotheses. These hypotheses are formulated to explore the connection between COVID-19 fear, COVID-19 stress and the health care performance of nurses. Regarding H1, we proposed a negative association between COVID-19 fear and the health care performance of nurses. The findings revealed that COVID-19 fear led toa decrease in the health care performance of nurses. The findings of the current study show the negative effect of COVID-19 fear on the health care performance of nurses. 

The results also revealed that studyH2 confirmed that COVID-19 fear significantly caused COVID-19 stress. It is suggested that COVID-19 fear is the leading force behind COVID-19 stress. Moreover, study H3 suggested a negative association between COVID-19 stress and the health care performance of nurses.

Regarding the H4results, it was confirmed that COVID-19 fear predicts the health care performance of nurses via COVID-19 stress. COVID-19 fear increases the stress that reduces the performance of individuals in the workplace. The findings revealed that COVID-19 fear regarding the COVID-19 pandemic became the cause of the stress which in turn negatively affected the performance of nurses due to heavy workloads and the numerous rescheduling of work activities. 

### 4.1. Theoretical Contribution 

The current study has some valuable theoretical contributions which contribute to the health care management research. This study marks a substantial addition in the prevailing understanding pertaining to the COVID-19 pandemic in an effective and distinguished manner. The main contributions of the study are that it is one of the exceptional studies that have tested COVID-19 fear as a determinant of COVID-19 stress and the performance of nursing staffs. There may hardly be any study which has made such a comprehensive explanation for the performance of nursing staffs.

Second, this study offers health care performance of nurses-Model for health care sector. This model demonstrates how the integrated elements e.g., COVID-19 fear and COVID-19 stress, affect the health care performance of nurses. 

Third, the strength of this investigation refers to the review of COVID-19 fear in affectingCOVID-19 stress. COVID-19 stress is an important psychological related issue and disorder of the individual regarding the worldwide pandemic. There has been limited deliberation on the role of COVID-19 stress with respect to its determinants and outcomes. Therefore, to fill this gap in the existing knowledge, this research focused on COVID-19 fear as a potential determinants of COVID-19 stress. 

### 4.2. Practical Implications

The current study also has valuable implications for practice. First, the current study suggested that health care organizations can enhance the performance of health care professionals with the help of reducing COVID-19 fear and stress among the professional workforce. By doing so, efficient performance can only be achieved when organization positively respond to the problems of the nursing staffs that merged due to the COVID-19 pandemic. Second, this study suggests that COVID-19 fear increased stress among the nursing staffs. Therefore, through proper facilities, work schedules and equipment, health care organizations can strengthen the health care performance of their nursing staffs. Furthermore, the management should consider the workloads of health care professionals, which is one of the distracting factors for reducing the performance of health care professionals. For the improvement of performance of health care professionals, management should provide a flexible working environment for better job performance in the workplace.

### 4.3. Limitations and Future Research

Beyond various theoretical and practical implications, the current research also has some limitations. First, the current study was based on a cross sectional design. However, a longitudinal design can be used for understanding the impact of COVID-19 on health care professionals’ performance. Second, the current study only considered stress among nurses as a mediating variable between COVID-19 fear and health care performance. In future studies, other psychological factors can also be used as mediators. Finally, the data for testing the hypothesized model was collected from the hospitals, therefore the findings are not generalize-able to other sectors. In future studies, this model can be tested in other sectors to understand the destructive role of COVID-19 fear.

## 5. Conclusions

The purpose of this study was to highlight the psychological factors that are the consequences of the COVID-19 pandemic. Fear is one of the important psychological concerns of the worldwide pandemic situation. COVID-19 fear is one of the psychological factors that cause various emotional illness among individuals worldwide. The most destructive aspect of COVID-19 fear is psychological strain, i.e., stress. The current study found negative effects of COVID-19 fear on both stress and the performance of nurses. Furthermore, findings also suggested that COVID-19 stress also negatively affects the performance in the workplace due to the occurrence of unusual events or actions. In summary we concluded that COVID-19 fear increases the stress among the nurses, which becomes the reason for their lower performance in the workplace.

## Figures and Tables

**Table 1:** Correlation.

Constructs	Mean	SD	1	2	3	4	5	6	7
Gender	0.9	0.81	1						
Age	33		0.09	1					
Work experience	2.9	0.84	0.08	0.03	1				
Education level	2.4	0.91	0.06	0.05	0.04	1			
COVID-19 Fear	3.8	0.93	0.09	0.12 *	0.08	0.07	1		
COVID-19 Stress	3.5	0.91	0.05	0.09	0.04	0.05	0.32 **	1	
HCP of nurses	3.9	0.95	0.03	0.07	0.06	0.09	−0.21 *	−0.30 **	1

Note: SD (Standard Deviation). * < 0.005 ** < 0.001.

**Table 2:** Validity Test.

	Item(s)	Alpha	FL	CR	A.V.E
COVID-19 Fear	7	0.82	0.73-0.91	0.82	0.68
COVID-19 Stress	07	0.81	0.70-0.88	0.80	0.71
HCP of Nurses	13	0.89	0.76-0.90	0.84	0.73

**Table 3:** Results for the mediating effects of EMS.

Variables	DV: HCP of Nurses		DV: COVID-19 Stress	
	M.1	M.2	M.3	M.4
Predictors				
COVID-19 fear	−0.23 **	−0.13		0.34 ***
COVID-19 Stress		−0.37 ***		
*R* ^2^	0.38	0.38	0.045	0.38
Adjusted *R*^2^	0.34	0.35	0.031	0.35
*F*-value	20.65 ***	49.86 **	3.10 **	37.64 ***
Durbin-Watson	2.096	2.014	1.823	2.215

*** = *p* < 0.000 and ** = *p* < 0.005.

## Data Availability

Data is kept highly confidential for best public interest and respondents.

## Supplementary Materials

The following are available online at https://www.mdpi.com/article/10.3390/nursrep11040087/s1. Table S1. STROBE Statement—checklist of items that should be included in reports of observational studies.

## Appendix A

COVID-19 Fear

1. I feel afraid due to COVID-19.
2. I feel uncomfortable when think about COVID-19.
3. My hands become damp when I think about COVID-19.
4. I become nervous or anxious When listening about COVID-19 on social media,
5. My life is disturbed due to COVID-19.
6. I cannot sleep due to fear of COVID-19.
7. My heart races when I think about getting COVID-19.

Health care performance of nurses

To what extent:

1. Availability of Vaccination causes to affect performance?
2. Availability of drugs causes to affect performance?
3. Availability of drugs (Anti-malarial), causes to affect performance?
4. Availability of drugs (Ferrous Sulphate) causes to affect performance.
5. Availability of equipment causes to affect performance?
6. Availability of electricity causes to affect performance?
7. Availability of water causes to affect performance?
8. Office buildings space influences you to work comfortably?
9. Privacy influences your interactions with patients.
10. Working tools causes to affect operating procedure.
11. You communicate with client
12. Feedback on findings of physical and other procedures done
13. Vaccination to the baby is important for Orient women.

COVID-19 Stress

How do you perceive:

1. the risk of contagion due to COVID-19 pandemic?
2. the condition of social isolation due to COVID-19 pandemic?
3. the relationships with your relatives due to COVID-19 pandemic?
4. the relationships with your colleagues due to COVID-19 pandemic?
5. the relationships with your health care professionals due to COVID-19 pandemic?
6. your performance experience due to COVID-19 pandemic?
7. the changes in your sexual life due to the social isolation due to COVID-19 pandemic?
